# A Comparison of Different Guidelines for the Treatment of Acute Heart Failure and Their Extensibility to Emergency Departments: A Critical Reappraisal

**DOI:** 10.3390/jcm14103522

**Published:** 2025-05-17

**Authors:** Lorenzo Falsetti, Emanuele Guerrieri, Vincenzo Zaccone, Silvia Santini, Laura Giovenali, Giulia Pierdomenico, Alessia Raponi, Linda Elena Gialluca Palma, Gianluca Moroncini, on behalf of the ITEMS Study Group

**Affiliations:** 1 Clinica Medica, Dipartimento di Scienze Cliniche e Molecolari, Università Politecnica delle Marche, 60126 Ancona, Italy; 2 Emergency Medicine Residency Program, Università Politecnica delle Marche, 60126 Ancona, Italy; 3 Internal and Subintensive Medicine, Azienda Ospedaliero-Universitaria delle Marche, 60126 Ancona, Italy; 4 Internal Medicine Residency Program, Università Politecnica delle Marche, 60126 Ancona, Italy

**Keywords:** acute heart failure, emergency department, guidelines, diuretics, inotropes, vasopressors, lung ultrasound, point-of-care ultrasound

## Abstract

**Background/Objectives:** Acute heart failure (AHF) is a common cause of emergency department (ED) referral. Different guidelines aim to optimise this condition, but the evidence is poor for most indications regarding AHF. In this paper, we aim to (i) identify the five most interesting topics in AHF management, (ii) compare guidelines, and (iii) give the reader the best advice on how to manage AHF in each topic. **Methods:** The working group (WG) identified ten critical topics in AHF management using a Delphi method and submitted them to ITEMS, a national group of ED physicians that ranked them. The WG selected the five highest-ranked topics and performed a critical reappraisal of guidelines. Lastly, the WG prepared the answers for ED physicians according to the guidelines and comparisons of studies. **Results:** The WG identified five topics for ED patients with AHF: (i) what is the optimal oxygen and ventilatory support, (ii) what is the optimal dose and administration modality of diuretics, (iii) what is the role of inotropes and vasopressors, (iv) what therapeutic strategies are suggested for diuretic refractoriness, and (v) what is the diagnostic role of lung ultrasound. For each topic, the WG identified, summarised, and compared the indications provided by each guideline, generating advice for ED management of AHF after a critical literature reappraisal. **Conclusions:** We highlighted the differences among guidelines dealing with AHF and developed the five best recommendations to assist ED physicians in determining the optimal approach for the ED management of AHF and to suggest future research directions.

## 1. Introduction

Acute heart failure (AHF) is a life-threatening medical emergency with a very wide spectrum of clinical manifestations, ranging from cardiogenic shock to mild congestion, that requires prompt diagnosis and intervention. It is a common cause of hospitalisation and is associated with high morbidity and mortality rates. Diagnosing and managing AHF in an emergency department (ED) is a complex and challenging task requiring a multidisciplinary approach. The initial management of AHF includes the early identification and correction of conditions presenting with acute hemodynamic compromise, particularly cardiogenic shock, acute coronary syndromes, arrhythmias, hypertensive crises, mechanical causes, pulmonary embolism, systemic infections, and cardiac tamponade [1]. The ED physician should promptly identify these diseases with clinical, electrocardiographic, and instrumental methods, with a particular interest in point-of-care ultrasound (PoCUS) [2]. Hemodynamically stable patients with suspected AHF should be diagnosed through a structured physical examination and an electrocardiogram (ECG). Organising this phase should be performed using validated pre-test assessment tools, such as the Brest or PREDICA score [3]. After assessing pre-test probability, the diagnosis should be confirmed through testing natriuretic peptide levels in patients with low pre-test probability or evaluated with a lung ultrasound (LUS) or chest X-ray in those with intermediate pre-test probability [2]. Patients with high pre-test probability may be treated directly, and an LUS or chest X-ray can help identify potential triggers of AHF, such as infections. AHF treatment in an ED should be tailored to the patient’s history, presentation, and underlying comorbidities. The main goals to achieve in this phase are to improve hemodynamics, reduce symptoms and congestion, and to prevent complications. The initial treatment routinely includes oxygen supplementation, ventilatory support with CPAP or NIV, diuretics, and vasodilators, whilst vasopressors and inotropic agents should be reserved for cardiogenic shock or hypoperfusion states. The management of AHF in an ED also involves identifying and managing precipitating factors such as infections, arrhythmias, and myocardial infarction. Antibiotics, anticoagulants, and antiarrhythmic agents can improve outcomes in these patients. Currently, the complex diagnostic and therapeutic management of heart failure (HF) is described in guidelines produced by various scientific societies, primarily reflecting the cardiologists’ perspective. These documents reflect an increasing interest in innovative targets and therapies designed to enhance survival rates, reduce re-hospitalisation frequency, and improve overall quality of life. For instance, particular attention has been directed towards sodium–glucose cotransporter inhibitors, neprilysin inhibitors, and the evaluation and correction of iron metabolism, which are generally managed by cardiologists during the hospitalisation phase [1,4,5,6,7]. Among the most cited guidelines, only one document is created by emergency physicians [8]. Furthermore, while the management of chronic and advanced HF—commonly handled by cardiologists—are well represented in almost all the guidelines, instructions on AHF diagnosis and treatment in an ED—which is crucial for emergency physicians—constitute only a minor section in most documents. Additionally, the diagnostic and therapeutic management of AHF in an ED varies significantly between countries, reflecting cultural and organisational differences. Guidelines from national or continental societies represent the local configuration of care for AHF [9]. This paper aims to select the most important guidelines on HF management, focusing on (i) identifying five key topics in AHF management through a Delphi method and national survey directed at an Italian emergency physician society, (ii) comparing guidelines on the five selected topics and their clinical questions, and (iii) providing the reader with the best advice for managing AHF in each topic after comparing guidelines and evidence.

## 2. Materials and Methods

We organised the methodology for this critical reappraisal of guidelines in the following four consecutive steps:

- *First step: Working group creation and guidelines selection:* The working group (WG) consisted of ten internal emergency medicine specialists who were working in emergency medicine departments and residents of the Emergency Medicine Residency Program of Marche Polytechnic University, Ancona, Italy. The WG identified the most cited and recent guidelines dealing with AHF management. In each guideline, the WG identified the suggested diagnostic or therapeutic indications and critically assessed the text, searching both the text and the cited references.
- *Second step: Delphi Rounds:* Each member of the WG analysed the selected guidelines, identifying the most critical topics pertinent to AHF diagnosis and treatment within an ED. The topics identified by each member were submitted anonymously to the other participants. After one month, the WG convened to assess the topics and prepared a document delineating the ten most salient topics concerning AHF management in an ED, as illustrated in Table 1.

- *Third step: National survey:* The list of topics was sent to Italian Emergency Medicine Schools (ITEMSs) members, using Google Forms as the method of distribution. The ITEMSs group consists of emergency medicine academics and residents or internal medicine specialists working in an emergency medicine setting. In the form, each ITEMSs member was asked to answer three general questions regarding (i) their current role (resident, academic, or consultant), (ii) their obtained or ongoing specialisation (emergency medicine, internal medicine, or other specialisation), and (iii) their place of work (ED as a resident, ED as a consultant, step-down unit as a resident, step-down unit as a consultant, or other). Then, the form asked each member of the ITEMSs group to rank each topic with a vote ranging from 1 (not interesting) to 5 (very interesting). The forms were anonymous. The WG selected the first five highly ranked indications for further consideration. The rank was determined by adding together the number of votes of “4” (interesting) and “5” (very interesting) for each question.
- *Fourth step: Guidelines comparison and critical reappraisal:* Each topic was divided into specific clinical questions. Firstly, the WG compared the indications for each question across guidelines in tables. Then, the WG critically analysed the sources used to generate the evidence in the selected guidelines. Finally, the WG synthesised the directions and generated specific answers for each question.

## 3. Results

*Guidelines Selection:* The WG identified the following guidelines as the most updated, accessed, and cited in the literature at the moment of the study: the 2021 European Society of Cardiology (ESC), the 2022 American Heart Association (AHA), the 2021 update on the 2014 guidelines of National Institute for Health and Excellence (NICE), the 2017 Canadian Cardiovascular Society (CCS), and the 2022 American College of Emergency Physicians (ACEP) guidelines [1,5,6,7,8]. The WG also considered the ESC 2023 update on HF treatment. However, since several trials considered by this update were not available at the moment of the publication of the other guidelines, we limited our comparison to the original guidelines, considering this document only to improve the discussion on the diuretic treatment chapter to offer the reader updated information [4].

*Grading of Evidence:* When analysing the guidelines, the WG observed a difference in the grading of evidence between guidelines, which makes a direct comparison of indications complex:

- *ESC guidelines* include four recommendation classes: (i) *Class I* is “recommended”; (ii) *Class II* is divided into IIa “should be considered” and IIb “may be considered”; and (iii) *Class III* is “not recommended”. The guidelines also categorise the following evidence levels: (i) *Level A* is from “multiple randomised trials or meta-analyses”; (ii) *Level B* from a “single randomised trial or large non-randomized studies”; and (iii) *Level C* is from expert consensus or small, retrospective studies and registries [1].
- *CCS and NICE guidelines* classify recommendation levels and evidence quality using the GRADE system as follows: (i) *High*, “further research unlikely to alter confidence in effect”; (ii) *Moderate*, “further research likely affects confidence and may change the estimate”; (iii) *Low*, “further research very likely impacts confidence and likely changes the estimate”; and (iv) *Very Low*, “effect estimate is very uncertain”. The GRADE system also provides two recommendation grades: (i) *Strong*, where “desirable effects clearly outweigh undesirable ones”, and (ii) *Weak*, where “trade-offs are uncertain due to low-quality evidence or closely balanced effects” [5,6].
- *AHA guidelines* classify recommendations into five categories: (i) *Class 1, or Strong,* which means the benefits greatly outweigh the risks, using terms like “is recommended” or “should be performed”; (ii) *Class 2a, or Moderate,* which means that the benefits moderately exceed risks, as indicated by “is reasonable”; (iii) *Class 2b, or Weak*, which means that the benefits slightly exceed or equal risks, with terms like “may/might be reasonable”; (iv) *Class 3, or No benefit*, which means that the benefits equal the risks; and (v) *Class 3, or Harm*, which indicates that the risks outweigh the benefits, using terms like “potentially harmful”. Additionally, the AHA classifies evidence levels as follows: (i) *Level A*, “high-quality evidence from multiple RCTs”; (ii) *Level B-R*, “moderate-quality evidence from RCTs”; (iii) *Level B-NR*, “moderate-quality evidence from non-randomised studies”; (iv) *Level C-LD*, “limited data from observational studies”; and (v) *Level C-EO*, “expert opinion based on clinical experience” [7].
- *ACEP Guidelines* recommendations have four categories: (i) *Class I*, “high-quality trials or meta-analyses”; (ii) *Class II*, “non-randomized trials and diagnosis, retrospective studies” (iii) *Class III*, “case series”; and (iv) *Class X*, “studies with significant limitations, which are not included in recommendations but may inform background consensus”. Evidence is graded into the following three levels: (i) *Level A*, “high-certainty patient care principles based on Class I or consistent Class II studies”; (ii) *Level B*, “moderate certainty recommendations from Class II or III studies”; and (iii) *Level C*, “Class III studies or expert consensus due to insufficient literature” [8].

*Results of the Delphi Rounds and Survey:* The WG selected ten topics at the end of the Delphi rounds, as shown in Table 1: these topics were sent via Google Forms to the ITEMSs group members. A total of 99 forms were returned, and of these 68.7% were submitted by residents while 31.3% were sent by consultants. Doctors responding to the form were most commonly residents or specialists in emergency medicine (81.8%) and were less frequently in internal medicine (14.1%) or other specialities (4%). Table 1 illustrates the topics selected by the WG and the number of votes cast by the sample of ITEMSs members. We combined questions 2 and 5 into a single domain to enhance the discussion.

*Topic 1: What is the optimal oxygen and ventilatory support for a patient with AHF in the ED?*

Of the five guidelines considered, the ESC and CCS guidelines address the question of oxygen treatment along with the indications for continuous positive airway pressure (CPAP), non-invasive ventilation (NIV), and invasive airway ventilation (IAV). The role of CPAP, NIV, and IAV is also discussed in the NICE guidelines, which do not address the question of oxygen therapy as the guideline development group emphasised that this treatment, in the context of AHF, does not differ from standard clinical practice [1,5,6]. The comparison among guidelines on this topic is presented in Table 2.

*Indications*

- *Oxygen:* Both guidelines agree on and assign a level of evidence for oxygen treatment in hypoxaemia, defined as oxygen saturation (SpO_2_) <90% or a partial oxygen pressure (PaO_2_) < 60 mmHg in blood gas analysis. In this group, neither guideline specifies a particular or comorbidity-adjusted SpO_2_ target after initiating oxygen: the CCS guidelines recommend achieving a SpO_2_ > 90%. Oxygen treatment is not recommended for normoxic AHF patients, and both guidelines emphasise the potential harm of excess oxygen without assigning a level of evidence for this indication.
- *CPAP or NIV:* Two of the three guidelines advise against the routine use of CPAP/NIV in AHF and assign a level of evidence against this practice, while the ESC guidelines do not address this issue. The three guidelines agree to use non-invasive methods only in specific subsets of patients; both the ESC and CCS guidelines recommend early CPAP/NIV use to alleviate dyspnea and reduce the risk of invasive ventilation in patients experiencing acute respiratory distress, a respiratory rate exceeding 25 breaths per minute, and persistent hypoxia with oxygen saturation below 90%. The ESC guidelines assign a level of evidence to this practice, while the CCS suggests this indication in the text. The NICE guidelines recommend considering CPAP or NIV for patients with severe dyspnea and acidemia, both at acute presentation and as an adjunct to medical therapy if their condition fails to improve.
- *Endotracheal intubation and mechanical ventilation:* This measure is described in all three guidelines as a rescue procedure; however, we observed slight discrepancies among the guidelines regarding the indications for IAV. The ESC guidelines suggest endotracheal intubation and mechanical ventilation for patients with respiratory insufficiency that persists despite oxygen or non-invasive ventilation, providing a level of evidence; the NICE guidelines recommend this intervention in cases of AHF complicated by non-responsive respiratory failure, reduced consciousness, or physical exhaustion, along with a level of evidence; and the CCS guidelines note that endotracheal intubation may be considered in instances of non-invasive ventilation failure or cardiogenic shock, without providing a level of evidence.

*Topics 2–5: What is the optimal dose and modality of diuretics for a patient with AHF in the ED? What therapeutic strategies are recommended for a patient with AHF who is refractory to diuretics in the ED?*

Four guidelines currently recommend intravenous diuretics as the first pharmacological approach for patients with AHF [1,5,6,7]. ACEP guidelines suggest early administration of diuretics but do not address the dosage and method of diuretic infusion [8].

We observed significant differences in the recommended starting dose and subsequent administration methods among the four guidelines. Similarly, there are minor discrepancies regarding the indication of thiazide and thiazide-like diuretics, low-dose inotropes, and ultrafiltration. A comparison of the guidelines is presented in Table 3.

*Indications*

- *Intravenous diuretics:* There is substantial agreement on intravenous diuretics use as the cornerstone of fluid overload treatment in AHF [1,5,6,7,8]. Loop diuretics are considered to be first-line treatments due to their pharmacokinetics and efficacy. Two guidelines endorse initiating the diuretic even in the pre-hospital phase [5,8]. There is no explicit consensus among the reviewed documents regarding the need to begin with a furosemide bolus, as suggested by the ESC guidelines. The guidelines agree that there is no difference in the infusion method, recommending continuous infusion or pulsed bolus. For loop diuretic dosing, a “low-dose” strategy is defined as the intravenous administration of the same dose as the oral equivalent, which is suggested as the initial approach by the ESC guidelines [1]. A “high-dose” strategy, characterised as the intravenous administration of 2.5 times the oral dose, is currently supported by the AHA, NICE, and CCS guidelines [5,6,7]. These discrepancies reflect varying interpretations of the results of the DOSE trial [10]. Also, there is disagreement among guidelines on the usefulness of an early furosemide administration. The ESC guidelines emphasise the lack of evidence for the optimal timing of loop diuretics, whereas the CCS and ACEP suggest that early administration should be considered as soon as AHF diagnosis is made [1,7,8].
- *Thiazide or thiazide-like diuretics:* In cases of resistant oedema or diuretic resistance, three guidelines (ESC, CCS, and AHA) recommend adding a thiazide or thiazide-like diuretic, while the NICE guidelines do not support this practice due to a lack of consistent evidence [1,5,6,7]. The efficacy of combination therapy involving thiazides, thiazide-like diuretics, or acetazolamide has also been addressed in the 2023 update of the ESC guidelines in light of more recent studies such as ADVOR and CLOROTIC [4]. However, the absence of an effect on major clinical outcomes, such as mortality, has prevented modifications to the recommendations established by the ESC [4].
- *Mineralocorticoid receptor antagonists:* Two guidelines (AHA and CCS) consider adding a mineralocorticoid receptor antagonist (MRA) to the diuretic strategy to facilitate decongestion in AHF [5,7].
- *Low-dose dopamine:* Inotropes and vasopressors are contraindicated by all guidelines in individuals without low cardiac output or cardiogenic shock. When diuretics alone or a combination of diuretics is insufficient to reduce congestion and promote weight loss, a “low-dose” dopamine strategy has been suggested and widely implemented in the past. Three guidelines address this topic, and while the CCS opposes this practice, the AHA and NICE guidelines indicate that further studies are necessary as some individuals appear to experience a degree of improvement.
- *Ultrafiltration:* No guidelines recommend ultrafiltration as an alternative to diuretics, particularly for patients with cardiorenal syndrome and deteriorating renal function. Two guidelines suggest considering ultrafiltration for refractory volume overload unresponsive to diuretic treatment. The AHA guidelines emphasise that the risks of ultrafiltration outweigh its benefits, without providing a definitive contraindication to its use, while the CCS guidelines advise against its use even in patients with diuretic-resistant oedema.

*Topic 3: What is the role of inotropes or vasopressors in patients with AHF in the ED?*

Of the five guidelines, ESC, NICE, AHA, and CCS deal with the topic of inotropes or vasopressors use in this setting [1,5,6,7]. As synthesised in Table 4, there is general consistency between documents on the indications of inotropes or vasopressors.

*Indications*

- *Routine inotropes or vasopressors administration in haemodynamically stable patients with AHF:* Three guidelines (ESC, NICE, and CCS) advise against the routine use of inotropes in haemodynamically stable patients with AHF [1,5,6].
- *Inotropes and vasopressors in haemodynamically unstable subjects:* In the setting of a patient with hypotension or low cardiac output, the ESC and CCS guidelines recommend inotropes to maintain end-organ perfusion and enhance cardiac performance, as well as vasopressors to improve perfusion in cases of cardiogenic shock. The AHA advises the use of inotropes solely for cardiogenic shock, while the NICE guidelines suggest either inotropes or vasopressors for potentially reversible cardiogenic shock (5,6).
- *Types of inotropes:* There is currently no consensus on the preferred inotrope to utilise in this clinical context. The ESC guidelines advocate for the use of levosimendan or type-3 phosphodiesterase inhibitors in preference to dobutamine for the risk of tachycardia and arrhythmias and suggest using these drugs, particularly in subjects treated with beta-blockers. The CCS guidelines recommend considering dobutamine in cases of cardiogenic shock and dobutamine or milrinone in low-output states. The AHA guidelines indicate that there is insufficient evidence to demonstrate a definitive advantage of one inotropic agent over another, asserting that the choice should be guided by the clinical presentation of the patient [1,5,7].
- *Types of vasopressors:* There is no consensus regarding the preferred vasopressor for shocked patients. The ESC guidelines advocate the use of norepinephrine over dopamine and epinephrine, emphasising the lesser efficacy and safety of these two drugs. The CCS guidelines indicate that dopamine or alternative vasopressors may be considered, while the AHA guidelines state that norepinephrine and epinephrine should be considered for cardiogenic shock [1,5,7].

*Topic 4: What is the role of lung ultrasound in the diagnosis of AHF in the ED?*

Despite the capabilities of lung ultrasounds, the AHA, NICE, and CCS guidelines do not acknowledge this method and instead focus the diagnosis on natriuretic peptides [5,6,7]. The ESC guidelines reference this technique among other first-line imaging studies to be considered for AHF diagnosis, emphasising the use of natriuretic peptides. This document further underscores that lung ultrasounds and chest X-rays may be utilised to confirm the diagnosis, mainly when natriuretic peptide testing is unavailable [1]. Only the ACEP 2022 guidelines recommend a lung ultrasound in the context of AHF and assign a level of evidence to this procedure, as illustrated in Table 5 [8]. The ACEP guidelines highlight improved diagnostic accuracy, shortened time to diagnosis, and quicker time to treatment for patients with AHF assessed via lung ultrasound, supporting these conclusions with several systematic reviews featuring meta-analyses. The ACEP guidelines assert that a diagnostic strategy incorporating a lung ultrasound is superior to other approaches based on a NT-proBNP or chest X-ray for identifying AHF in cases of undifferentiated dyspnoea [11]. Furthermore, this document specifies that a lung ultrasound reduced diagnostic errors in 8% of patients and decreased the median time to diagnosis from 104.5 to 5 min [8,11].

## 4. Discussion

The survey underlined that the population of residents and consultants working in Italian EDs were mainly interested in the following five specific domains: oxygen and ventilatory support, point-of-care LUS as a diagnostic method, diuretic treatment in the acute phase, decongestion strategies in refractory oedema, and inotropes or vasopressors use.

Of note, the most voted question regarded LUS use as a diagnostic method in an ED, which is the most under-represented topic in the considered guidelines. LUS, however, is commonly used in an ED as the first diagnostic method for AHF.

When we analysed the current guidelines on the selected topics, we observed a general agreement on the analysed domains, with some differences that may reflect the different sanitary systems, as in the case of the AHA and ESC guidelines, and the different specialists involved in guideline production, as in the case of the ACEP guidelines which are made by emergency medicine physicians.

According to our revision, the selected topics and their relative questions should be answered as follows to guide the ED management of a patient with AHF:

*Topic 1: What is the optimal oxygen and ventilatory support for a patient with AHF in the ED?*

- *There is no indication for oxygen supplementation in normoxic AHF subjects:* The supposed detrimental effects of hyperoxia on cardiac function are supported by older studies considering only small cohorts of subjects with AHF with reduced ejection fraction or chronic HF. These preliminary observations suggested that hyperoxia could worsen myocardial relaxation with an increased filling pressure and a further deterioration of cardiac function with a significant reduction in stroke volume and cardiac output [11,12,13,14]. However, recent clinical studies observed no effects of PaO_2_ levels on clinical outcomes in AHF [15], with no benefits of a lower SpO_2_ target on NT-pro-BNP levels, dyspnea, and clinical outcomes [16]. Critically ill patients with AHF and hyperoxemia did not show an increased risk of death, in-hospital stay, or need for mechanical ventilation [17,18,19]. In one study, adding oxygen to normoxic AHF subjects was associated with a longer in-hospital stay [17]. Thus, while oxygen supplementation has a defined role in hypoxemic patients, further studies should clarify its effects on normoxic patients [11,20].
- *Oxygen should be offered to hypoxic AHF patients to correct hypoxemia*: Two of the considered guidelines agree against the routine use of oxygen therapy in non-hypoxemic subjects with AHF, supporting its use only in patients with SpO_2_ < 90% or PaO_2_ < 60 mmHg, without setting a specific SpO_2_ target, as shown in Table 2 [1,5]. The indication of oxygen supplementation for hypoxemic subjects is extrapolated from clinical practice and observational studies in the absence of placebo-controlled studies [21,22].
- *CPAP or NIV should not be started routinely in AHF subjects:* It must be emphasised that in the 3CPO trial most patients had little or no respiratory insufficiency and the mean PaO_2_ was 100 mmHg, supporting the absence of indication for non-invasive ventilation methods in AHF patients without respiratory failure [23].
- *CPAP and NIV should be started early in all AHF patients with respiratory failure:* Three guidelines agree against the routine use of CPAP or NIV in AHF, supporting this practice only in subjects with evidence of acute respiratory failure, as shown in Table 2, with a low level of evidence [1,5,6]. The largest randomised trial, the 3CPO study, showed no benefit in terms of mortality at 7 and 30 days and no differences in intubation rates when comparing NIV or CPAP with standard medical treatment [23]. However, several meta-analyses based on older observational studies demonstrated the efficacy of NIV and CPAP in reducing in-hospital mortality and the need for intubation, especially in acute cardiogenic pulmonary oedema (ACPOE) with respiratory failure [24,25,26,27]. The beneficial effect of using positive pressure was particularly pronounced in patients whose AHF event was due to an ischaemic myocardial cause [24]. A recent Cochrane meta-analysis confirmed the efficacy of CPAP or NIV in reducing in-hospital mortality and intubation rates during ACPOE [28]. Thus, CPAP or NIV are currently suggested by guidelines and consensus statements in the setting of AHF with respiratory failure, especially in the case of ACPOE [29]. Of note, evidence suggests that early, prehospital CPAP use seems to be associated with better outcomes in this setting [30,31].
- *Invasive airway management should be considered only in AHF patients with respiratory failure who do not improve with non-invasive ventilation or with absolute contraindications to non-invasive ventilation*: Three guidelines give an indication for orotracheal intubation for progressive respiratory failure not responding to oxygen or non-invasive ventilation [1,6]. The NICE AHF guidelines also suggest orotracheal intubation in subjects with reduced consciousness and signs of physical exhaustion [6]. These indications are in line with clinical practice and the literature as patients directly treated with orotracheal intubation show worse outcomes than those treated with a trial of non-invasive ventilation and, in case of failure, orotracheal intubation [32].

*Topics 2–5: What is the optimal dose and modality of diuretics for a patient with AHF in the ED? What therapeutic strategies are recommended for a patient with AHF who is refractory to diuretics in the ED?*

- *Loop diuretics should be used early in all AHF patients, and an initial iv bolus should be considered:* All of the guidelines agree on the use of loop diuretics as first-line treatment to reduce congestion in AHF [1,5,6,7,8]. However, we observed significant differences in the indications from the analysed guidelines for loop diuretic doses and administration modalities. A loading furosemide bolus should be considered to reach the threshold concentration faster to invoke natriuresis [33]. Administering loop diuretics early appears reasonable once a diagnosis of AHF with volume overload is made. Studies assessing the effectiveness of administering diuretics in the prehospital phase or reducing door-to-diuretic time in the ED are not univocal, with some indicating minimal or no benefit in decreasing the length of stay or in-hospital mortality and others showing some degree of benefit [34,35,36,37]. An ED physician should not delay an accurate differential diagnosis of acute undifferentiated dyspnoea and administer diuretics early to those who will truly benefit from this intervention.
- *Loop diuretic dose should be personalised according to the patient’s clinical presentation, diuretic response and comorbidities:* The DOSE trial did not find any significant difference between a high-dose regimen (2.5 times the oral dose) and a low-dose regimen (1 time the oral dose) of furosemide in symptom relief or serum creatinine levels [38]. However, a high-dose regimen was associated with a more significant reduction in dyspnea and weight, thus, it was recommended by the AHA and CCS guidelines [5,7]. In contrast, the ESC 2021 guidelines suggest a low-dose strategy to reduce the neurohormonal activation associated with high-dose regimens, which is deemed to increase mortality in AHF [1,39]. The NICE and ACEP guidelines do not formulate an indication regarding the optimal diuretic dose [6,8]. An ED physician should consider the clinical presentation, the natriuretic response to the initial loop diuretic bolus, and the patient’s comorbidities to tailor the optimal diuretic dose. A high-dose regimen should be considered for patients with marked congestion signs and symptoms or with comorbidities suggesting a potentially reduced response to low-dose regimens.
- *There is no preferred loop diuretic administration modality between boluses and continuous infusion:* The considered guidelines, according to the published trials, do not formulate an indication for a modality of one administration over another [1,5,6,7,8]. A recent Cochrane systematic review confirmed insufficient data to suggest a specific modality of loop diuretic delivery [40]. Continuous infusions, however, do not allow for the achievement of high furosemide doses. Patients responding only to high doses, as in the case of diuretic resistance, could be managed with pulsed boluses and a multi-agent drug strategy to pierce the minimum threshold of diuretic efficacy [41].
- *Consider the association with thiazide or thiazide-like diuretics in subjects with a reduced response to loop diuretics:* A multi-drug strategy in AHF is currently suggested by the ESC and AHA and contemplated by the CCS guidelines, especially for patients with resistant oedema or diuretic resistance, while the NICE guidelines do not formulate a recommendation due to the low methodological quality of the studies in this field [1,5,6,7]. The most studied diuretics in this setting are hydrochlorothiazide and metolazone and their association with loop diuretics within 24 h from admission for AHF has been associated with an increased volume loss, but also with increased rates of worsening renal function and a non-significant improvement of death, length of stay, and the re-hospitalisation rate [42,43,44]. The CARRESS-HF trial underlined that a stepped pharmacological algorithm considering thiazide and thiazide-like diuretics was as effective as ultrafiltration in reducing congestion, with fewer side effects [44]. Acetazolamide showed similar efficacy in improving the reduction in congestion in AHF, with no observed effects on mortality reduction and increased rates of worsening renal failure; thus, it is still not considered by guidelines as a potential pharmacological approach [1,4,45]. Nevertheless, it is essential to emphasise that one of the objectives of an ED physician, particularly during the initial hours, is to alleviate congestion and enhance diuresis. Achieving this outcome is often facilitated by the combination of two or more classes of diuretics, as evidenced by both historical and contemporary studies. Consequently, particularly in patients who do not exhibit an adequate natriuretic response to high-dose regimens of loop diuretics, implementing combination strategies may be considered to enhance diuresis and alleviate systemic and pulmonary congestion.
- *MRA should be added early in the context of guideline-directed therapy, but has little or no effect on reducing congestion in AHF:* None of the considered guidelines suggests adding MRA in the acute phase of HF with a level of evidence [1,5,6,7,8]. However, the AHA and CCS guidelines mention in the text the possible use of spironolactone, as shown in Table 3 [5,7]. In the ATHENA-HF trial, spironolactone failed to show any improvement in NT-proBNP levels, urine output, weight changes, symptoms, or congestion in the first 96 h [46,47]. This effect can be associated with the peculiar spironolactone pharmacokinetics, as being a prodrug its clinical effects can be observed only after 72 h from the administration; thus, the efficacy of steroidal MRA in an ED to reduce congestion is limited due to their slow mechanism of action. However, MRA addition may reduce hypokalaemia and contraction alkalosis in patients undergoing large-volume diuresis. More recent studies suggest that early, in-hospital MRA initiation improves long-term outcomes: MRA are part of the HF guideline-directed medical therapy and should be initiated early during hospitalisation according to the most recent trials [48,49]. Less information is available on non-steroidal MRA, eplerenone and finerenone. However, preliminary studies have shown interesting results [50].
- *Do not offer ultrafiltration routinely to AHF patients; ultrafiltration should be reserved for highly selected patients with congestion who are unresponsive to diuretic strategies:* There is no agreement between guidelines on the role of ultrafiltration in AHF. According to the CARESS-HF trial, ultrafiltration seems inferior to a stepped pharmacological treatment in reducing congestion and serum creatinine levels, with more serious adverse events [44]. Other studies have shown improved outcomes, significant weight loss, and fluid removal among the patients treated with ultrafiltration [51,52]. However, a recent systematic review with meta-analysis underlined that ultrafiltration could reduce readmission rates, while the effect on all-cause mortality is less clear [53]. Moreover, ultrafiltration does not seem superior to diuretics on dyspnea, clinical status, weight loss, and renal function, with an increased risk of long-term renal replacement therapy [6,53]. Thus, further studies are still required to clarify the role of ultrafiltration in AHF; at the moment of this revision, it could be considered among selected subjects unresponsive to stepped diuretic strategies according to the ESC and NICE guidelines, while the CCS statement does not recommend this practice at all [1,5,6]. The AHA guidelines underline the uncertainty of this intervention and do not give any indication nor contraindication to ultrafiltration in AHF [7]. From a real-world perspective, an ED physician should acknowledge the significance of a stepped diuretic algorithm and its non-inferiority to ultrafiltration during the acute phase of the illness, thereby limiting this practice to the small percentage of non-responsive patients admitted to intensive care units.

*Topic 3: What is the role of inotropes and vasopressors in patients with AHF in the ED?*

- *Do not routinely offer inotropes or vasopressors in patients with AHF:* Four guidelines agree on the indication against the routine use of inotropes in AHF, as shown in Table 4 [1,5,6,7]. In the absence of high-quality studies addressing this topic in AHF, the indication to avoid the routine administration of short-term inotropes or vasopressors is generated by expert opinion and negative results from CHF studies, registries, and small AHF trials that showed an increased risk of arrhythmias, myocardial infarction, and in-hospital death [54].
- *Do not offer low-dose inotropes or vasopressors to improve diuresis in haemodynamically stable patients with AHF:* Four guidelines give a clear indication against use of inotropes or vasopressors in haemodynamically stable patients [1,5,6,7]. However, while the NICE guidelines state that further trials are required to assess the role of low-dose inotropes or vasopressors in increasing decongestion, AHA and CCS recommend against this practice, as shown in Table 4 [5,6,7]. Several studies, such as ROSE-AHF, DAD-HF, and DAD-HF II, have already analysed the efficacy of low-dose inotropes, particularly dopamine, to increase diuresis and promote decongestion when compared with placebo and higher or lower furosemide doses [10,55,56]: these trials demonstrated no advantage of dopamine compared to a placebo in decongestion or mortality. Instead, they revealed an increased rate of adverse events, such as atrial fibrillation and tachycardia.
- *Inotropes or vasopressors can be considered only in AHF patients with hypotension and signs of end-organ hypoperfusion or in the setting of cardiogenic shock:* Four guidelines agree on the indication of inotrope or vasopressor treatment in subjects with AHF and hypoperfusion with end-organ damage or low cardiac output, with different levels of evidence, as shown in Table 5 [1,5,6,7,57]. Vasopressors should be considered to maintain pressure, while inotropes should be preferred to improve cardiac output. Both categories are useful for maintaining end-organ perfusion. Several studies have found an increased risk of death associated with both inotropes and vasopressor use in AHF [58,59,60]. However, in the context of AHF with a low cardiac output phenotype, the use of these agents often represents the last therapeutic option, albeit supported by a very low quality of evidence [58]. Several trials, such as OPTIME-HF, CAPITAL-DOREMI, and SURVIVE, assessed their efficacy in AHF survival, underlining no significant differences between different inotropic drugs and between inotropes and a placebo [61,62,63,64,65]. A recent guideline focused on inotropic agents use for patients with acute circulatory failure did not recommend one drug over another in the setting of cardiogenic shock, underlining the very low quality of evidence and the substantial similarity between inotropes and placebos in short-term and middle-term outcomes [66]. Studies with limited sample sizes support vasopressor use in this clinical context. The rationale primarily stems from meta-analyses that indicate the superiority of norepinephrine in both safety and efficacy when compared to other drugs, especially epinephrine [67,68,69,70,71]. There is no evidence to support dopamine use in this setting: among the other studies, the SOAP-II trial showed a significant increase in mortality and arrhythmias in the setting of patients affected by cardiogenic shock and treated with this specific drug [67].

*Topic 4: What is the role of lung ultrasound in the diagnosis of AHF in the ED?*

- *Lung ultrasound should be considered a first-line diagnostic method for AHF in the ED:* Despite the common, real-life use in EDs, only two guidelines consider LUS as a potential method to diagnose AHF; ESC 2021 mentions this method in the text, but only the ACEP guidelines provide a degree of evidence for this practice [1,8]. LUS is commonly used to diagnose AHF in undifferentiated dyspneic patients, and several meta-analyses have focused on the overall diagnostic value of LUS in AHF, confirming the good specificity and sensibility of this method and underlining that its performance improves when integrated with an inferior vena cava and echocardiographic assessment [2,72,73,74]. LUS is more sensitive than chest radiography in subjects assessed in the ED for dyspnea due to AHF, with a similar specificity [75,76]. Moreover, integrating LUS with other point-of-care ultrasound techniques can help the clinician to perform an accurate differential diagnosis in the dyspneic subject [77]. The ACEP guidelines underline that a LUS-based approach improves diagnostic accuracy, while natriuretic peptides and chest X-rays do not [8,75,78]. Moreover, the CEP guidelines enlighten the concept that LUS allows a faster diagnosis, and thus, a faster time-to-treatment, which has been associated with better outcomes in AHF [8]. This practice is currently suggested to improve diagnosis in EDs by an Italian consensus of the most influential scientific societies, including cardiology, emergency medicine, and internal medicine doctors [79]. Thus, LUS use for AHF diagnosis in EDs should be suggested and encouraged [80].

## 5. Conclusions

The comparison between the most influential and cited guidelines on AHF highlights several, albeit minor, differences in managing patients with AHF. These discrepancies should be interpreted within the framework of different healthcare systems, available resources, and social and cultural distinctions. Furthermore, the heterogeneity of studies conducted over an extensive timeframe, as referenced in these documents, must also be acknowledged by the reader as contributing to this diversity. By comparing the guidelines and critically reappraising the selected indications, we aimed to suggest some “best practice” indications for ED physicians from selected domains, as synthesised in Table 6.

## Figures and Tables

**Table 1:** Results of the Delphi rounds and ranking of the selected topics after the national survey.

Rank	Question	Votes (*n*, %)
1	What is the role of lung ultrasound in diagnosing AHF in the ED?	94 (94.9%)
2	What therapeutic strategies are recommended for a patient with AHF who is refractory to diuretics in the ED?	92 (92.9%)
3	What is the optimal oxygen and ventilatory support for a patient with AHF in the ED?	88 (88.9%)
4	What is the role of inotropes and vasopressors in patients with AHF in the ED?	87 (87.9%)
5	What is the optimal dose and modality of diuretic infusion for a patient with AHF in the ED?	85 (85.8%)
6	Which evidence supports the clinical phenotypization of the AHF patient in the ED?	77 (77.7%)
7	What is the role of clinical stratification rulers (EHMRG, MEESSI-AHF) in managing AHF in the ED?	71 (71.7%)
8	What is the role of natriuretic peptides in diagnosing AHF in the ED?	56 (56.6%)
9	What is the role of mechanical supports for the circulation in the ED?	52 (52.5%)
10	What is the role of natriuretic peptides in diagnosing and managing AHF in the ED?	44 (43.4%)

*Legend:* AHF: acute heart failure; ED: emergency department.

**Table 2:** What is the optimal oxygen and ventilatory support for a patient with AHF in the ED?

	ESC	AHA	NICE	CCS	ACEP
Routine use of oxygen supplementation	Not suggested	N/A	N/A	Not Suggested	N/A
	**Text:** “*In AHF, oxygen should not be used routinely in non-hypoxaemic patients, as it causes vasoconstriction and a reduction in cardiac output”* **LoE:** *N/A*			**Text:** “*This recom-mendation places higher value on the physiologic studies showing potential harm with the use of excess oxygen in normoxic patients and less value on the long-term clinical usage of supplemental oxygen without supportive data”* **LoE:** *N/A*	
Oxygen supplement in case of hypoxemia	Suggested	N/A	Suggested	Suggested	N/A
	**Text:** *“Oxygen is recommended in patients with SpO_2_ < 90% or PaO_2_ < 60 mmHg to correct hypoxaemia”.* **LoE:** *IC*		**Text:** *“The GDG agreed that it was not necessary to make a recom-mendation on the use of supplementary oxygen as an alternative method of ventilatory support as its usage is standard practice”* **LoE:** *N/A*	**Text:** *“We recommend supplemental oxygen be considered for patients who are hypoxemic; titrated to an oxygen saturation > 90%”* **LoE:** *Strong Recommendation; Moderate-Quality Evidence*	
Routine use of CPAP or NIV	N/A	N/A	Not Suggested	Not Suggested	N/A
			**Text:** *“Do not routinely use non-invasive ventilation (continuous positive airway pressure [CPAP] or non-invasive positive pressure ventilation [NIPPV]) in people with acute heart failure and cardiogenic pulmonary oedema”* **LoE:** *From High to Very Low*	**Text:** *“We recommend that CPAP or bilevel positive airway pressure (BiPAP) not be used routinely in/for patients with AHF”* **LoE:** *Strong Recommendation; Moderate-Quality Evidence*	
Use of CPAP or non-invasive positive pressure support in case of respiratory failure and hypoxemia	Suggested	N/A	Suggested	Suggested	N/A
	**Text:** *“Non-invasive positive pressure ventilation should be considered in patients with respiratory distress (respiratory rate > 25 breaths/min, SpO_2_ < 90%) and started as soon as possible in order to decrease respiratory distress and reduce the rate of mechanical endotracheal intubation”* **LoE:** *IIaB*		**Text:** *“If a person has cardiogenic pulmonary oedema with severe dyspnoea and acidaemia consider starting non-invasive ventilation without delay: at acute presentation or as an adjunct to medical therapy if the person’s condition has failed to respond”* **LoE:** *From High to Very Low*	**Text:** *“Treatment with BiPAP/CPAP might be appropriate for patients* *with persistent hypoxia (peripheral O2 saturation < 90%),* *high respiratory rate (>25 breaths per minute), and* *pulmonary edema despite other appropriate therapies”.* **LoE:** *N/A*	
Invasive ventilation in patients in respiratory distress or respiratory failure despite oxygen or non-invasive positive pressure support	Suggested	N/A	Suggested	Suggested	N/A
	**Text:** *“Intubation is recommended for progressive respiratory failure persisting in spite of oxygen administration or non-invasive ventilation”* **LoE:** *IC*		**Text:** *“Consider invasive ventilation in people with acute heart failure that, despite treatment, is leading to or is complicated by respiratory failure or reduced consciousness or physical exhaustion”* **LoE:** *Very Low*	**Text:** *“Endotracheal intubation might be used if less invasive modes of oxygen delivery fail* *or if the patient is in cardiogenic shock”* **LoE:** *N/A*	

*Legend:* ACEP: American College of Emergency Physicians; AHA: American Heart Association; CCS: Canadian Cardiovascular Society; ESC: European Society of Cardiology; LoE: level of evidence; and N/A: not addressed. We have highlighted in bold the caption for the text and the level of evidence and indicated in italics the original sentence taken from the guidelines.

**Table 3:** What is the optimal dose and modality of diuretics for a patient with AHF in the ED? What therapeutic strategies are recommended for a patient with AHF who is refractory to diuretics in the ED?

	ESC	AHA	NICE	CCS	ACEP
Intravenous diuretics for all patients with AHF	Suggested	Suggested	Suggested	Suggested	N/A
	**Text:** *“Intravenous loop diuretics are recommended for all patients with AHF admitted with signs/symptoms of fluid overload to improve symptoms”* **LoE:** *IC*	**Text:** *“Patients with HF admitted with evidence of significant fluid overload should be promptly treated with intravenous loop diuretics to improve symptoms and reduce morbidity”* **LoE:** *1 B-NR*	**Text:** *“Offer intravenous diuretic therapy to people with acute heart failure”* **LoE:** *From Moderate to Very Low*	**Text:** *“We recommend that I.V. diuretics be given as first-line therapy for patients with pulmonary or peripheral congestion”* **LoE:** *Strong Recommendation; Low-Quality Evidence*	
Type of loop diuretic: Furosemide Torasemide Bumetanide	Suggested Suggested Suggested	Suggested Suggested Suggested	Suggested Suggested Suggested	Suggested N/A N/A	N/A
Early loop diuretics administration	Uncertain	N/A	N/A	Suggested	Suggested
	**Text:** *“Data defining their optimal dosing, timing, and method of administration are limited”* **LoE:** *N/A*			**Text:** *“Diuretic therapy may be initiated in the ambulance, HF clinic, or in-hospital”* **LoE:** *N/A*	**Text:** *“Although no specific timing of diuretic therapy can be recommended, physicians may consider earlier administration of diuretics when indicated for emergency department patients with acute heart failure syndrome, because it may be associated with reduced length of stay and inhospital mortality”* **LoE:** *Level C*
Loop diuretics administration: pulse versus infusion strategy	No differences	No differences	No differences	No differences	N/A
	**Text:***“Daily single bolus administrations* *are discouraged because of the possibility of post-dosing sodium retention. With continuous infusion, a loading dose may be used to achieve steady state earlier”* **LoE:** *N/A*	**Text:** *“In the DOSE (Diuretic Optimization Strategies Evaluation) trial, there were no significant differences in patients’ global assessment of symptoms or in the change in renal function when diuretic therapy was administered by bolus, compared with continuous infusion or at a high dose compared with a low dose”* **LoE:** *N/A*	**Text:** *“start treatment using either a bolus or infusion strategy”* **LoE:** *From Moderate to Very Low*	**Text:** *“We recommend that for patients requiring I.V. diuretic therapy, furosemide may be dosed intermittently (eg, twice daily) or as a continuous infusion”* **LoE:** *Strong Recommendation; Moderate-Quality Evidence*	
High loop diuretic dose versus low loop diuretic dose	Low dose	High dose	High dose	High dose	N/A
	**Text:** *“Based on these observations, it may be appropriate, when starting i.v. diuretic treatment, to use low doses, to assess the diuretic response and increase the dose when that is insufficient”* **LoE:** *N/A*	**Text:** “*In patients hospitalized with HF when diuresis is inadequate to relieve symptoms and signs of congestion, it is reasonable to intensify the diuretic regimen using either: a. higher doses of intravenous loop diuretics or addition of a second diuretic”* **LoE:** *2a B-NR*	**Text:** *“For people already taking a diuretic, consider a higher dose of diuretic than that on which the person was admitted unless there are serious concerns with patient adherence to diuretic therapy before admission”* **LoE*:*** *From Moderate to Very Low*	**Text:** *“Thus, there is no advantage in the routine use of continuous diuretic infusions and a higher dose of diuretics could be considered for many* *patients, with careful observation of renal function and electrolytes”.* **LoE:** *N/A*	
Combination with thiazide or thiazide-like diuretic	Suggested	Suggested	Not Suggested	Can be considered	N/A
	**Text:** *“Combination of a loop diuretic with thiazide-type diuretic should be considered in patients with resistant oedema who do not respond to an increase in loop diuretic doses”* **LoE:** *IIaB*	**Text:** “*In patients hospitalized with HF when diuresis is inadequate to relieve symptoms and signs of congestion, it is reasonable to intensify the diuretic regimen using either: a. higher doses of intravenous loop diuretics or addition of a second diuretic”* **LoE:** *2a B-NR*	**Text:** *“However, there is some inconsistent and non-robust evidence that addition of a thiazide or thiazide-like diuretic (metolazone) may be beneficial”* **LoE:** *N/A*	**Text:***“Combining loop diuretics with thiazides or spironolactone has been proposed and appears effective, with fewer side effects than a higher dose of a* *loop diuretic”.* **LoE:** *N/A*	
Combination with MRA	N/A	Suggested	N/A	Can be considered	N/A
		**Text:** *“Titration to achieve effective diuresis may require doubling of initial doses, adding a thiazide diuretic, or adding an MRA that has diuretic effects in addition to its cardiovascular benefits”* **LoE:** *B-NR*		**Text:***“Combining loop diuretics with thiazides or spironolactone has been proposed and appears effective, with fewer side effects than a higher dose of a* *loop diuretic”.* **LoE:** *N/A*	
Low-dose dopamine to improve diuresis in normotensive AHF	N/A	Uncertain	Uncertain	Not Suggested	N/A
		**Text:** *“Addition of low-dose dopamine to diuretic therapy in the setting of reduced eGFR did not improve outcomes in a study that included patients with all EFs, but a subset analysis showed increased urine output and weight loss in patients with LVEF < 0.40”* **LoE:** *N/A*	**Text:** *“The GDG considered that low dose dopamine might be appropriate to assist diuresis in certain patients, but recognised that a stronger evidence base was required, so recommended a further trial is carried out to address this question”* **LoE:** *N/A*	**Text:** *“Low-dose dopamine has been studied in the context of AHF and does not improve clinical symptoms, renal function, or reduce clinical events”.* **LoE:** *N/A*	
Ultrafiltration as an alternative to diuretics	N/A	Uncertain	Not Suggested	N/A	N/A
		**Text:** “*Many aspects of ultrafiltration including patient selection, fluid removal rates, venous access, prevention of therapy-related complications, and cost require further investigation”* **LoE:** *N/A*	**Text:** *“Do not routinely offer ultrafiltration to people with acute heart failure”* **LoE:** *From Moderate to Very Low*		
Ultrafiltration in the case of unresponsive oedema	Suggested	Uncertain	Suggested	Not Suggested	N/A
	**Text:** *“Ultra-filtration may be considered in refractory volume overload unresponsive to diuretic treatment”* **LoE:** *IIbC*	**Text:** “*Many aspects of ultrafiltration including patient selection, fluid removal rates, venous access, prevention of therapy-related complications, and cost require further investigation”* **LoE:** *N/A*	**Text:** *“Consider ultrafiltration for people with confirmed diuretic resistance”* **LoE:** *From Moderate to Very Low*	**Text:** *“We do not recommend the routine use of ultrafiltration (UF) for the management of intractable edema in decompensated HF“* **LoE:** *Weak Recommendation; Low-Quality Evidence*	

*Legend:* ACEP: American College of Emergency Physicians; AHA: American Heart Association; AHF: acute heart failure; CCS: Canadian Cardiovascular Society; ESC: European Society of Cardiology; LoE: level of evidence; MRA: mineralocorticoid receptor antagonist; and N/A: not addressed. We have highlighted in bold the caption for the text and the level of evidence and indicated in italics the original sentence taken from the guidelines.

**Table 4:** What is the role of inotropes or vasopressors in patients with AHF in the ED?

	ESC	AHA	NICE	CCS	ACEP
Inotropes for all patients with AHF	Not Suggested	N/A	Not Suggested	Not Suggested	N/A
	**Text:** *“Inotropic agents are not recommended routinely, due to safety concerns, unless the patient* *has symptomatic hypotension and evidence of* *hypoperfusion”.* **LoE:** *IIIC*		**Text:** *“Do not routinely offer inotropes or vasopressors to people with acute heart failure”* **LoE:** *Very Low*	**Text:** *“We recommend that hemodynamically stable patients not routinely receive inotropes like dobutamine, dopa-mine, levosimendan, or milrinone”* **LoE:** *Strong Recommendation; High-Quality Evidence*	
Inotropes and vasopressors in patients with AHF, hypotension, and signs of end-organ hypoperfusion	Suggested	Suggested	Can be considered	Can be considered	N/A
	**Text:** *“Inotropic agents may be considered in patients with SBP < 90 mmHg and evidence of hypoperfusion who do not respond to standard treatment,* *including fluid challenge, to improve peripheral* *perfusion and maintain end-organ function”* **LoE:** *IIbC* **Text:** *“A vaso-pressor, preferably norepinephrine, may be considered in patients with cardiogenic shock to increase blood pressure and vital organ perfusion”* **LoE:** *IIbB*	**Text:** *“In patients with cardiogenic shock, intravenous* *inotropic support should be used to maintain* *systemic perfusion and preserve end-organ performance”* **LoE:** *1 B-NR*	**Text:** *“Consider inotropes or vasopressors in people with acute heart failure with potentially reversible cardiogenic shock”.* **LoE:** *Very Low*	**Text:** *“In patients with low SBP (<90 mmHg), low cardiac output and either euvolemia or hypervolemia,* *inotropes may be used for stabilization”* **LoE:** *N/A* **Text:** *“I.V. vasoconstrictor agents (eg, phenylephrine, norepinephrine) should generally be avoided for AHF* *management except for hypotensive patients with SBP < 90* *mm Hg, associated signs or symptoms, end-organ damage,* *and a significant change from baseline”* **LoE:** *N/A*	
	Suggested	Suggested	N/A	Suggested	N/A
Types of inotropes	**Text:***“Inotropes, especially those with adrenergic mechanisms, can cause sinus tachycardia, increase ventricular rate in patients with AF, may induce myocardial ischaemia and arrhythmias, and increase mortality. Levosimendan or type-3-phosphodiesterase inhibitors may be preferred over dobutamine for patients on beta-blockers as they act through independent mechanisms. Excessive peripheral vasodilation and hypotension can be major limitations of type-3-phosphodiesterase inhibitors or levosimendan, especially when administered at high doses and/or when commenced with a bolus dose”.* **LoE:** *N/A*	**Text:***“Intravenous inotropic support can increase cardiac output and improve hemodynamics in patients presenting with cardiogenic shock. Despite their ubiquitous use for initial management of cardiogenic shock, there are few prospective data and a paucity of randomised trials to guide their use. However, their broad availability, ease of administration, and clinician familiarity favour such agents as the first therapeutic consideration when signs of organ hypoperfusion persist despite empiric volume replacement and vasopressors. There is a lack of robust evidence to suggest the clear benefit of one intronic agent over another in cardiogenic shock. In general, the choice of a specific inotropic agent is guided by blood pressure, concurrent arrhythmias, and availability of drug”* **LoE:** *N/A*		**Figure 9, Page 1368 in CCS guidelines:** *“Consider dobutamine in patients in shock, consider dobutamine or milrinone in subjects with low cardiac output”* **Text: *“*** *Inotropic agents have not been shown to improve patient* *outcomes. The Outcomes of a Prospective Trial of* *Intravenous Milrinone for Exacerbations of Chronic Heart* *Failure (OPTIME-CHF) trial randomized 951 patients* *admitted for HF to a 48-h infusion of milrinone or pla* *cebo. New-onset atrial arrhythmias, worsening HF, and* *symptomatic hypotension requiring intervention occurred* *more frequently in the milrinone group. A nonsignificant* *increase in the number of deaths in-hospital and after 60 days* *was seen in the milrinone group. A post hoc analysis showed a* *higher incidence of death or rehospitalization in patients with* *underlying ischemic HF etiology. Trials using levosimendan have not shown additional benefit compared with* *placebo; omecamtiv mecarbil is undergoing further testing* *in a RCT. Low-dose dopamine has been studied in the* *context of AHF and does not improve clinical symptoms, renal function, or reduce clinical events”.* **LoE:** N/A	
	Suggested	Suggested	N/A	Suggested	N/A
Types of vasopressors	**Text:** *“Among drugs with a prominent peripheral arterial vasoconstrictor* *action, norepinephrine may be preferred in patients with severe* *hypotension. The aim is to increase perfusion to the vital organs.* *However, this is at the expense of an increase in LV afterload.* *Therefore, a combination of norepinephrine and inotropic agents may be considered, especially in patients with advanced HF and cardiogenic shock. Some studies, though with limitations, support the use of norepinephrine as first choice, compared with dopamine or epinephrine. Dopamine was compared with norepinephrine as a first-line vasopressor therapy in patients with shock and was associated with more arrhythmic events and with a greater mortality inpatients with cardiogenic shock”* *“In another prospective randomized trial epinephrine was compared with norepinephrine in patients with cardiogenic shock due to acute MI. The trial was stopped prematurely due to a higher incidence of refractory shock with epinephrine”* **LoE:** N/A	**Table 20, page e958 in AHA guideline:** *“Vasopressors: Norepinephrine and Epinephrine”* **LoE:** N/A		**Figure 9, Page 1368 in CCS guidelines:** *“Consider dopamine or other vasopressor in shock”* **LoE:** N/A	

*Legend:* ACEP: American College of Emergency Physicians; AHA: American Heart Association; AHF: acute heart failure; CCS: Canadian Cardiovascular Society; ESC: European Society of Cardiology; LoE: level of evidence; MRA: mineralocorticoid receptor antagonist; and N/A: not addressed. We have highlighted in bold the caption for the text and the level of evidence and indicated in italics the original sentence taken from the guidelines.

**Table 5:** What is the role of lung ultrasound in diagnosing AHF in the ED?

	ESC	AHA	NICE	CCS	ACEP
LUS for AHF diagnosis	Can be considered	N/A	N/A	N/A	Indicated
	**Text:** *“Additional investigations, i.e., chest X-ray and lung ultrasound may be used to confirm AHF diagnosis, especially when NP testing is not available”.* **LoE:** N/A				**Text:** *“Use point-of-care lung ultrasound as an imaging modality in conjunction with medical history and physical examination to diagnose acute heart failure syndrome when diagnostic uncertainty exists as the accuracy of this diagnostic test is sufficient to direct clinical management”.* **LoE:** Level B

*Legend:* ACEP: American College of Emergency Physicians; AHA: American Heart Association; AHF: acute heart failure; CCS: Canadian Cardiovascular Society; ESC: European Society of Cardiology; LoE: level of evidence; MRA: mineralocorticoid receptor antagonist; N/A: not addressed. We have highlighted in bold the caption for the text and the level of evidence and indicated in italics the original sentence taken from the guidelines.

**Table 6:** Summary of the indications obtained from the critical reappraisal of HF guidelines.

Summary of the Indications Obtained from the Critical Reappraisal
Topic 1: Which is the optimal oxygen and ventilatory support for a patient with AHF in the ED? There is no indication for oxygen supplementation in normoxic AHF subjects. Oxygen should be offered to hypoxic AHF patients to correct hypoxemia. CPAP or NIV should not be started routinely in AHF subjects. CPAP and NIV should be started early in all AHF patients with respiratory distress or acidemia. Invasive airway management should be considered only in AHF patients with respiratory failure who do not improve with non-invasive ventilation or with absolute contraindications to non-invasive ventilation.
Topic 2–5: Which is the optimal dose and modality of diuretics for a patient with AHF in the ED? Loop diuretics should be used early in all AHF patients, and an initial iv bolus should be considered. Loop diuretic dose should be personalised according to the patient’s clinical presentation, diuretic response, and comorbidities. There is no preferred furosemide administration modality between boluses and continuous infusion. Consider the association with thiazide or thiazide-like diuretics in subjects with a reduced response to loop diuretics. MRA should be added early in the context of guideline-directed therapy but it has little or no effect on reducing congestion in AHF. Do not offer ultrafiltration routinely to AHF patients; ultrafiltration should be reserved for carefully selected patients with congestion who are unresponsive to diuretic strategies.
Topic 3: What is the role of inotropes and vasopressors in patients with AHF in the ED? Do not routinely offer inotropes or vasopressors to patients with AHF. Do not offer low-dose inotropes or vasopressors to improve diuresis in haemodynamically stable patients with AHF. Inotropes or vasopressors can be considered only in AHF patients with hypotension and signs of end-organ hypoperfusion or in the setting of cardiogenic shock.
Topic 4: What is the role of lung ultrasound in the diagnosis of AHF in the ED? Lung ultrasound should be considered a first-line diagnostic method for AHF in an ED.

## Data Availability

No new data were generated by this study.

## References

[1] McDonagh T.A., Metra M., Adamo M., Gardner R.S., Baumbach A., Böhm M., Burri H., Butler J., Čelutkienė J., Chioncel O. (2021). 2021 ESC Guidelines for the Diagnosis and Treatment of Acute and Chronic Heart Failure. Eur. Heart J..

[2] Martindale J.L., Wakai A., Collins S.P., Levy P.D., Diercks D., Hiestand B.C., Fermann G.J., de Souza I., Sinert R. (2016). Diagnosing Acute Heart Failure in the Emergency Department: A Systematic Review and Meta-analysis. Acad. Emerg. Med..

[3] Basset A., Nowak E., Castellant P., Gut-Gobert C., Le Gal G., L’Her E. (2016). Development of a Clinical Prediction Score for Congestive Heart Failure Diagnosis in the Emergency Care Setting: The Brest Score. Am. J. Emerg. Med..

[4] Filippatos G., Angermann C.E., Cleland J.G.F., Lam C.S.P., Dahlström U., Dickstein K., Ertl G., Hassanein M., Hart K.W., Lindsell C.J. (2020). Global Differences in Characteristics, Precipitants, and Initial Management of Patients Presenting With Acute Heart Failure. JAMA Cardiol..

[5] Heidenreich P.A., Bozkurt B., Aguilar D., Allen L.A., Byun J.J., Colvin M.M., Deswal A., Drazner M.H., Dunlay S.M., Evers L.R. (2022). 2022 AHA/ACC/HFSA Guideline for the Management of Heart Failure: A Report of the American College of Cardiology/American Heart Association Joint Committee on Clinical Practice Guidelines. Circulation.

[6] NICE National Institute for Health and Care Excellence Acute Heart Failure: Diagnosis and Management Clinical Guideline. https://www.nice.org.uk/guidance/cg187.

[7] Ezekowitz J.A., O’Meara E., McDonald M.A., Abrams H., Chan M., Ducharme A., Giannetti N., Grzeslo A., Hamilton P.G., Heckman G.A. (2017). 2017 Comprehensive Update of the Canadian Cardiovascular Society Guidelines for the Management of Heart Failure. Can. J. Cardiol..

[8] Silvers S.M., Gemme S.R., Hickey S., Mattu A., Haukoos J.S., Diercks D.B., Wolf S.J. (2022). Clinical Policy: Critical Issues in the Evaluation and Management of Adult Patients Presenting to the Emergency Department With Acute Heart Failure Syndromes: Approved by ACEP Board of Directors, June 23, 2022. Ann. Emerg. Med..

[9] McDonagh T.A., Metra M., Adamo M., Gardner R.S., Baumbach A., Böhm M., Burri H., Butler J., Čelutkienė J., Chioncel O. (2023). 2023 Focused Update of the 2021 ESC Guidelines for the Diagnosis and Treatment of Acute and Chronic Heart Failure. Eur. Heart J..

[10] Triposkiadis F.K., Butler J., Karayannis G., Starling R.C., Filippatos G., Wolski K., Parissis J., Parisis C., Rovithis D., Koutrakis K. (2014). Efficacy and Safety of High Dose versus Low Dose Furosemide with or without Dopamine Infusion: The Dopamine in Acute Decompensated Heart Failure II (DAD-HF II) Trial. Int. J. Cardiol..

[11] Sepehrvand N., Ezekowitz J.A. (2016). Oxygen Therapy in Patients With Acute Heart Failure. JACC Heart Fail..

[12] Park J.H., Balmain S., Berry C., Morton J.J., McMurray J.J.V. (2010). Potentially Detrimental Cardiovascular Effects of Oxygen in Patients with Chronic Left Ventricular Systolic Dysfunction. Heart.

[13] Mak S., Azevedo E.R., Liu P.P., Newton G.E. (2001). Effect of Hyperoxia on Left Ventricular Function and Filling Pressures in Patients With and Without Congestive Heart Failure. Chest.

[14] Haque W.A., Boehmer J., Clemson B.S., Leuenberger U.A., Silber D.H., Sinoway L.I. (1996). Hemodynamic Effects of Supplemental Oxygen Administration in Congestive Heart Failure. J. Am. Coll. Cardiol..

[15] Miñana G., Núñez J., Bañuls P., Sanchis J., Núñez E., Robles R., Mascarell B., Palau P., Chorro F.J., Llàcer A. (2011). Prognostic Implications of Arterial Blood Gases in Acute Decompensated Heart Failure. Eur. J. Intern. Med..

[16] Sepehrvand N., Alemayehu W., Rowe B.H., McAlister F.A., van Diepen S., Stickland M., Ezekowitz J.A. (2019). High vs. Low Oxygen Therapy in Patients with Acute Heart Failure: HiLo-HF Pilot Trial. ESC Heart Fail..

[17] Yu Y., Yao R.-Q., Zhang Y.-F., Wang S.-Y., Xi W., Wang J.-N., Huang X.-Y., Yao Y.-M., Wang Z.-N. (2021). Is Oxygen Therapy Beneficial for Normoxemic Patients with Acute Heart Failure? A Propensity Score Matched Study. Mil. Med. Res..

[18] Nael J., Ruggiu M., Bailleul C., Ortuno S., Diehl J.-L., Vimpère D., Augy J.-L., Guerot E., Danchin N., Puymirat E. (2019). Impact of Hyperoxia on Patients Hospitalized in an Intensive Care Unit for Acute Heart Failure. Arch. Cardiovasc. Dis..

[19] Chu M.Y.S., Guo W., Lim K.K., Lim B.L. (2020). Effect of Oxygen Therapy on the Risk of Mechanical Ventilation in Emergency Acute Pulmonary Edema Patients. Eur. J. Emerg. Med..

[20] Thomas A., van Diepen S., Beekman R., Sinha S.S., Brusca S.B., Alviar C.L., Jentzer J., Bohula E.A., Katz J.N., Shahu A. (2022). Oxygen Supplementation and Hyperoxia in Critically III Cardiac Patients. JACC Adv..

[21] O’Driscoll B.R., Howard L.S., Earis J., Mak V. (2017). BTS Guideline for Oxygen Use in Adults in Healthcare and Emergency Settings. Thorax.

[22] Helms J., Catoire P., Abensur Vuillaume L., Bannelier H., Douillet D., Dupuis C., Federici L., Jezequel M., Jozwiak M., Kuteifan K. (2024). Oxygen Therapy in Acute Hypoxemic Respiratory Failure: Guidelines from the SRLF-SFMU Consensus Conference. Ann. Intensive Care.

[23] Gray A., Goodacre S., Newby D.E., Masson M., Sampson F., Nicholl J. (2008). Noninvasive Ventilation in Acute Cardiogenic Pulmonary Edema. N. Engl. J. Med..

[24] Weng C.L., Zhao Y.T., Liu Q.H., Fu C.J., Sun F., Ma Y.L., Chen Y.W., He Q.Y. (2010). Meta-Analysis: Noninvasive Ventilation in Acute Cardiogenic Pulmonary Edema. Ann. Intern. Med..

[25] Winck J.C., Azevedo L.F., Costa-Pereira A., Antonelli M., Wyatt J.C. (2006). Efficacy and Safety of Non-Invasive Ventilation in the Treatment of Acute Cardiogenic Pulmonary Edema—A Systematic Review and Meta-Analysis. Crit. Care.

[26] Masip J., Roque M., Sánchez B., Fernández R., Subirana M., Expósito J.A. (2005). Noninvasive Ventilation in Acute Cardiogenic Pulmonary Edema. JAMA.

[27] Peter J.V., Moran J.L., Phillips-Hughes J., Graham P., Bersten A.D. (2006). Effect of Non-Invasive Positive Pressure Ventilation (NIPPV) on Mortality in Patients with Acute Cardiogenic Pulmonary Oedema: A Meta-Analysis. Lancet.

[28] Berbenetz N., Wang Y., Brown J., Godfrey C., Ahmad M., Vital F.M., Lambiase P., Banerjee A., Bakhai A., Chong M. (2019). Non-Invasive Positive Pressure Ventilation (CPAP or Bilevel NPPV) for Cardiogenic Pulmonary Oedema. Cochrane Database Syst. Rev..

[29] Masip J., Peacock W.F., Price S., Cullen L., Martin-Sanchez F.J., Seferovic P., Maisel A.S., Miro O., Filippatos G., Vrints C. (2018). Indications and Practical Approach to Non-Invasive Ventilation in Acute Heart Failure. Eur. Heart J..

[30] Foti G., Sangalli F., Berra L., Sironi S., Cazzaniga M., Rossi G.P., Bellani G., Pesenti A. (2009). Is Helmet CPAP First Line Pre-Hospital Treatment of Presumed Severe Acute Pulmonary Edema?. Intensive Care Med..

[31] Ducros L., Logeart D., Vicaut E., Henry P., Plaisance P., Collet J.-P., Broche C., Gueye P., Vergne M., Goetgheber D. (2011). CPAP for Acute Cardiogenic Pulmonary Oedema from Out-of-Hospital to Cardiac Intensive Care Unit: A Randomised Multicentre Study. Intensive Care Med..

[32] Tallman T.A., Peacock W.F., Emerman C.L., Lopatin M., Blicker J.Z., Weber J., Yancy C.W. (2008). Noninvasive Ventilation Outcomes in 2,430 Acute Decompensated Heart Failure Patients: An ADHERE Registry Analysis. Acad. Emerg. Med..

[33] Mullens W., Damman K., Harjola V., Mebazaa A., Brunner-La Rocca H., Martens P., Testani J.M., Tang W.H.W., Orso F., Rossignol P. (2019). The Use of Diuretics in Heart Failure with Congestion—A Position Statement from the Heart Failure Association of the European Society of Cardiology. Eur. J. Heart Fail..

[34] Matsue Y., Damman K., Voors A.A., Kagiyama N., Yamaguchi T., Kuroda S., Okumura T., Kida K., Mizuno A., Oishi S. (2017). Time-to-Furosemide Treatment and Mortality in Patients Hospitalized With Acute Heart Failure. J. Am. Coll. Cardiol..

[35] Wong Y.W., Fonarow G.C., Mi X., Peacock W.F., Mills R.M., Curtis L.H., Qualls L.G., Hernandez A.F. (2013). Early Intravenous Heart Failure Therapy and Outcomes among Older Patients Hospitalized for Acute Decompensated Heart Failure: Findings from the Acute Decompensated Heart Failure Registry Emergency Module (ADHERE-EM). Am. Heart J..

[36] Park J.J., Kim S.-H., Oh I.-Y., Choi D.-J., Park H.-A., Cho H.-J., Lee H.-Y., Cho J.-Y., Kim K.H., Son J.-W. (2018). The Effect of Door-to-Diuretic Time on Clinical Outcomes in Patients With Acute Heart Failure. JACC Heart Fail..

[37] Miró Ò., Harjola P., Rossello X., Gil V., Jacob J., Llorens P., Martín-Sánchez F.J., Herrero P., Martínez-Nadal G., Aguiló S. (2021). The FAST-FURO Study: Effect of Very Early Administration of Intravenous Furosemide in the Prehospital Setting to Patients with Acute Heart Failure Attending the Emergency Department. Eur. Heart J. Acute Cardiovasc. Care.

[38] Felker G.M., Lee K.L., Bull D.A., Redfield M.M., Stevenson L.W., Goldsmith S.R., LeWinter M.M., Deswal A., Rouleau J.L., Ofili E.O. (2011). Diuretic Strategies in Patients with Acute Decompensated Heart Failure. N. Engl. J. Med..

[39] ter Maaten J.M., Martens P., Damman K., Dickstein K., Ponikowski P., Lang C.C., Ng L.L., Anker S.D., Samani N.J., Filippatos G. (2020). Higher Doses of Loop Diuretics Limit Uptitration of Angiotensin-Converting Enzyme Inhibitors in Patients with Heart Failure and Reduced Ejection Fraction. Clin. Res. Cardiol..

[40] Rasoul D., Zhang J., Farnell E., Tsangarides A.A., Chong S.C., Fernando R., Zhou C., Ihsan M., Ahmed S., Lwin T.S. (2024). Continuous Infusion versus Bolus Injection of Loop Diuretics for Acute Heart Failure. Cochrane Database Syst. Rev..

[41] Jentzer J.C., DeWald T.A., Hernandez A.F. (2010). Combination of Loop Diuretics With Thiazide-Type Diuretics in Heart Failure. J. Am. Coll. Cardiol..

[42] Cox Z.L., Hung R., Lenihan D.J., Testani J.M. (2020). Diuretic Strategies for Loop Diuretic Resistance in Acute Heart Failure. JACC Heart Fail..

[43] Trullàs J.C., Morales-Rull J.L., Casado J., Carrera-Izquierdo M., Sánchez-Marteles M., Conde-Martel A., Dávila-Ramos M.F., Llácer P., Salamanca-Bautista P., Pérez-Silvestre J. (2023). Combining Loop with Thiazide Diuretics for Decompensated Heart Failure: The CLOROTIC Trial. Eur. Heart J..

[44] Bart B.A., Goldsmith S.R., Lee K.L., Givertz M.M., O’Connor C.M., Bull D.A., Redfield M.M., Deswal A., Rouleau J.L., LeWinter M.M. (2012). Ultrafiltration in Decompensated Heart Failure with Cardiorenal Syndrome. N. Engl. J. Med..

[45] Mullens W., Dauw J., Martens P., Verbrugge F.H., Nijst P., Meekers E., Tartaglia K., Chenot F., Moubayed S., Dierckx R. (2022). Acetazolamide in Acute Decompensated Heart Failure with Volume Overload. N. Engl. J. Med..

[46] Butler J., Anstrom K.J., Felker G.M., Givertz M.M., Kalogeropoulos A.P., Konstam M.A., Mann D.L., Margulies K.B., McNulty S.E., Mentz R.J. (2017). Efficacy and Safety of Spironolactone in Acute Heart Failure. JAMA Cardiol..

[47] Greene S.J., Felker G.M., Giczewska A., Kalogeropoulos A.P., Ambrosy A.P., Chakraborty H., DeVore A.D., Fudim M., McNulty S.E., Mentz R.J. (2019). Spironolactone in Acute Heart Failure Patients With Renal Dysfunction and Risk Factors for Diuretic Resistance: From the ATHENA-HF Trial. Can. J. Cardiol..

[48] Beldhuis I.E., Damman K., Pang P.S., Greenberg B., Davison B.A., Cotter G., Gimpelewicz C., Felker G.M., Filippatos G., Teerlink J.R. (2023). Mineralocorticoid Receptor Antagonist Initiation during Admission Is Associated with Improved Outcomes Irrespective of Ejection Fraction in Patients with Acute Heart Failure. Eur. J. Heart Fail..

[49] Mebazaa A., Davison B., Chioncel O., Cohen-Solal A., Diaz R., Filippatos G., Metra M., Ponikowski P., Sliwa K., Voors A.A. (2022). Safety, Tolerability and Efficacy of up-Titration of Guideline-Directed Medical Therapies for Acute Heart Failure (STRONG-HF): A Multinational, Open-Label, Randomised, Trial. Lancet.

[50] Kobayashi M., Yamashina A., Satomi K., Tezuka A., Ito S., Asakura M., Kitakaze M., Ferreira J.P. (2024). Adverse Events Associated with Early Initiation of Eplerenone in Patients Hospitalized for Acute Heart Failure. Int. J. Cardiol..

[51] Bart B.A., Boyle A., Bank A.J., Anand I., Olivari M.T., Kraemer M., Mackedanz S., Sobotka P.A., Schollmeyer M., Goldsmith S.R. (2005). Ultrafiltration Versus Usual Care for Hospitalized Patients With Heart Failure. J. Am. Coll. Cardiol..

[52] Costanzo M.R., Guglin M.E., Saltzberg M.T., Jessup M.L., Bart B.A., Teerlink J.R., Jaski B.E., Fang J.C., Feller E.D., Haas G.J. (2007). Ultrafiltration Versus Intravenous Diuretics for Patients Hospitalized for Acute Decompensated Heart Failure. J. Am. Coll. Cardiol..

[53] Srivastava M., Harrison N., Caetano A.F.S., Tan A.R., Law M. (2022). Ultrafiltration for Acute Heart Failure. Cochrane Database Syst. Rev..

[54] Bayram M., De Luca L., Massie M.B., Gheorghiade M. (2005). Reassessment of Dobutamine, Dopamine, and Milrinone in the Management of Acute Heart Failure Syndromes. Am. J. Cardiol..

[55] Chen H.H., Anstrom K.J., Givertz M.M., Stevenson L.W., Semigran M.J., Goldsmith S.R., Bart B.A., Bull D.A., Stehlik J., LeWinter M.M. (2013). Low-Dose Dopamine or Low-Dose Nesiritide in Acute Heart Failure With Renal Dysfunction. JAMA.

[56] Giamouzis G., Butler J., Starling R.C., Karayannis G., Nastas J., Parisis C., Rovithis D., Economou D., Savvatis K., Kirlidis T. (2010). Impact of Dopamine Infusion on Renal Function in Hospitalized Heart Failure Patients: Results of the Dopamine in Acute Decompensated Heart Failure (DAD-HF) Trial. J. Card. Fail..

[57] Follath F., Cleland J., Just H., Papp J., Scholz H., Peuhkurinen K., Harjola V., Mitrovic V., Abdalla M., Sandell E.-P. (2002). Efficacy and Safety of Intravenous Levosimendan Compared with Dobutamine in Severe Low-Output Heart Failure (the LIDO Study): A Randomised Double-Blind Trial. Lancet.

[58] Bloom J.E., Chan W., Kaye D.M., Stub D. (2023). State of Shock: Contemporary Vasopressor and Inotrope Use in Cardiogenic Shock. J. Am. Heart Assoc..

[59] Basir M.B., Lemor A., Gorgis S., Taylor A.M., Tehrani B., Truesdell A.G., Bharadwaj A., Kolski B., Patel K., Gelormini J. (2022). Vasopressors Independently Associated with Mortality in Acute Myocardial Infarction and Cardiogenic Shock. Catheter. Cardiovasc. Interv..

[60] Jentzer J.C., Wiley B., Bennett C., Murphree D.H., Keegan M.T., Kashani K.B., Bell M.R., Barsness G.W. (2020). Temporal Trends and Clinical Outcomes Associated with Vasopressor and Inotrope Use in The Cardiac Intensive Care Unit. Shock.

[61] Cuffe M.S. (2002). Short-Term Intravenous Milrinone for Acute Exacerbation of Chronic Heart Failure. A Randomized Controlled Trial. JAMA.

[62] Mathew R., Di Santo P., Jung R.G., Marbach J.A., Hutson J., Simard T., Ramirez F.D., Harnett D.T., Merdad A., Almufleh A. (2021). Milrinone as Compared with Dobutamine in the Treatment of Cardiogenic Shock. N. Engl. J. Med..

[63] Metra M., Nodari S., D’Aloia A., Muneretto C., Robertson A.D., Bristow M.R., Dei Cas L. (2002). Beta-Blocker Therapy Influences the Hemodynamic Response to Inotropic Agents in Patients with Heart Failure. J. Am. Coll. Cardiol..

[64] Mebazaa A., Nieminen M.S., Filippatos G.S., Cleland J.G., Salon J.E., Thakkar R., Padley R.J., Huang B., Cohen-Solal A. (2009). Levosimendan vs. Dobutamine: Outcomes for Acute Heart Failure Patients on Β-blockers in SURVIVE†. Eur. J. Heart Fail..

[65] Packer M., Colucci W., Fisher L., Massie B.M., Teerlink J.R., Young J., Padley R.J., Thakkar R., Delgado-Herrera L., Salon J. (2013). Effect of Levosimendan on the Short-Term Clinical Course of Patients With Acutely Decompensated Heart Failure. JACC Heart Fail..

[66] Møller M.H., Granholm A., Junttila E., Haney M., Oscarsson-Tibblin A., Haavind A., Laake J.H., Wilkman E., Sverrisson K.Ö., Perner A. (2018). Scandinavian SSAI Clinical Practice Guideline on Choice of Inotropic Agent for Patients with Acute Circulatory Failure. Acta Anaesthesiol. Scand..

[67] De Backer D., Biston P., Devriendt J., Madl C., Chochrad D., Aldecoa C., Brasseur A., Defrance P., Gottignies P., Vincent J.-L. (2010). Comparison of Dopamine and Norepinephrine in the Treatment of Shock. N. Engl. J. Med..

[68] Léopold V., Gayat E., Pirracchio R., Spinar J., Parenica J., Tarvasmäki T., Lassus J., Harjola V.-P., Champion S., Zannad F. (2018). Epinephrine and Short-Term Survival in Cardiogenic Shock: An Individual Data Meta-Analysis of 2583 Patients. Intensive Care Med..

[69] Levy B., Clere-Jehl R., Legras A., Morichau-Beauchant T., Leone M., Frederique G., Quenot J.-P., Kimmoun A., Cariou A., Lassus J. (2018). Epinephrine Versus Norepinephrine for Cardiogenic Shock After Acute Myocardial Infarction. J. Am. Coll. Cardiol..

[70] Myburgh J.A., Higgins A., Jovanovska A., Lipman J., Ramakrishnan N., Santamaria J. (2008). A Comparison of Epinephrine and Norepinephrine in Critically Ill Patients. Intensive Care Med..

[71] The TRIUMPH Investigators* (2007). Effect of Tilarginine Acetate in Patients With Acute Myocardial Infarction and Cardiogenic Shock. JAMA.

[72] Russell F.M., Ehrman R.R., Cosby K., Ansari A., Tseeng S., Christain E., Bailitz J. (2015). Diagnosing Acute Heart Failure in Patients With Undifferentiated Dyspnea: A Lung and Cardiac Ultrasound (LuCUS) Protocol. Acad. Emerg. Med..

[73] Anderson K.L., Jenq K.Y., Fields J.M., Panebianco N.L., Dean A.J. (2013). Diagnosing Heart Failure among Acutely Dyspneic Patients with Cardiac, Inferior Vena Cava, and Lung Ultrasonography. Am. J. Emerg. Med..

[74] Long B., Koyfman A., Gottlieb M. (2019). Diagnosis of Acute Heart Failure in the Emergency Department: An Evidence-Based Review. West. J. Emerg. Med..

[75] Pivetta E., Goffi A., Lupia E., Tizzani M., Porrino G., Ferreri E., Volpicelli G., Balzaretti P., Banderali A., Iacobucci A. (2015). Lung Ultrasound-Implemented Diagnosis of Acute Decompensated Heart Failure in the ED. Chest.

[76] Maw A.M., Hassanin A., Ho P.M., McInnes M.D.F., Moss A., Juarez-Colunga E., Soni N.J., Miglioranza M.H., Platz E., DeSanto K. (2019). Diagnostic Accuracy of Point-of-Care Lung Ultrasonography and Chest Radiography in Adults With Symptoms Suggestive of Acute Decompensated Heart Failure. JAMA Netw. Open.

[77] Mojoli F., Bouhemad B., Mongodi S., Lichtenstein D. (2019). Lung Ultrasound for Critically III Patients. Am. J. Respir. Crit. Care Med..

[78] Pivetta E., Goffi A., Nazerian P., Castagno D., Tozzetti C., Tizzani P., Tizzani M., Porrino G., Ferreri E., Busso V. (2019). Lung Ultrasound Integrated with Clinical Assessment for the Diagnosis of Acute Decompensated Heart Failure in the Emergency Department: A Randomized Controlled Trial. Eur. J. Heart Fail..

[79] Mortara A., Gabrielli D., Pugliese F.R., Corcione A., Perticone F., Fontanella A., Mercuro G., Cricelli C., Iacoviello M., D’Ambrosio G. (2019). ANMCO/FADOI/SIAARTI/SIC/SIMG/SIMI/SIMEU Consensus Document: The Clinical Care Pathway of Acute Heart Failure Patients from Symptom Onset to Discharge from the Emergency Department. G. Ital. Cardiol..

[80] Volpicelli G., Elbarbary M., Blaivas M., Lichtenstein D.A., Mathis G., Kirkpatrick A.W., Melniker L., Gargani L., Noble V.E., Via G. (2012). International Evidence-Based Recommendations for Point-of-Care Lung Ultrasound. Intensive Care Med..

